# Monoclonal light chain crystalline podocytopathy and tubulopathy associated with monoclonal gammopathy of renal significance: a case report and literature review

**DOI:** 10.1186/s12882-018-1108-x

**Published:** 2018-11-12

**Authors:** Xiao-juan Yu, Xu-jie Zhou, Su-xia Wang, Fu-de Zhou, Ming-hui Zhao

**Affiliations:** 1 Renal Division, Department of Medicine, Peking University First Hospital, Beijing, 100034 People’s Republic of China; 2 Institute of Nephrology, Peking University, Beijing, 100034 People’s Republic of China; 3 Key Laboratory of Renal Disease, Ministry of Health of China, Beijing, 100034 People’s Republic of China; 4 Key Laboratory of CKD Prevention and Treatment, Ministry of Education of China, Beijing, 100034 People’s Republic of China; 5 Laboratory of Electron Microscopy, Pathological Centre, Peking University First Hospital, Beijing, 100034 People’s Republic of China; 6 Peking-Tsinghua Center for Life Sciences, Beijing, 100871 People’s Republic of China

**Keywords:** Monoclonal gammopathy, MGRS, Crystal deposition, Podocytopathy

## Abstract

**Background:**

Monoclonal gammopathy of renal significance (MGRS) is a recently defined group of renal diseases caused by monoclonal immunoglobulin secreted by nonmalignant proliferative B cell or plasma cell. Monoclonal immunoglobulin can form different types of structures deposited in renal tissue, including fibrils, granules, microtubules, crystals and casts, and has mostly been reported in multiple myeloma patients. Here we report a rare case with κ light chain crystals in both podocytes and tubular epithelial cells associated with MGRS, which adds more information to the spectrum of MGRS-related renal diseases.

**Case presentation:**

A 53-year old woman presented with albumin–predominant moderate proteinuria and renal failure. She had monoclonal IgGκ in the serum and monoclonal IgGκ plus free κ in the urine. Multiple myeloma and lymphoproliferative disorders were excluded. Renal biopsy confirmed κ-restricted crystal-storing renal disease involving the podocytes and proximal tubular epithelial cells. The patient was treated with bortezomib followed by lenalidomide-based chemotherapy, and renal function was stable after 1 year of follow-up.

**Conclusions:**

This is a rare case of combined crystalline podocytopathy and tubulopathy associated with MGRS, in which diagnosis was dependent on electron and immuno-electron microscopy.

## Background

Monoclonal gammopathy of renal significance (MGRS) represents a group of renal diseases caused by direct deposition or indirect functional interference of monoclonal immunoglobulin (MIg), which is secreted by clonal B cells or plasma cells [1, 2]. Patients with MGRS do not meet the criteria for symptomatic multiple myeloma (MM) or lymphoma, the hematological abnormality is generally consistent with monoclonal gammopathy of undetermined significance (MGUS). However, the renal prognosis of MGRS is not benign.

The spectrum of MGRS includes a variety of renal lesions, among which renal amyloidosis, monoclonal immunoglobulin deposition disease, and cast nephropathy are the common types, whereas light chain proximal tubulopathy and crystal storing-histiocytosis, which are characterized by cytoplasmic crystallization of monoclonal light chains, are quite rare. Crystallization of MIgs can result in intravascular and/or intracellular crystal deposition, which has been reported mostly in MM [3–13] and rarely in MGRS [14, 15]. Here, we report a case of crystal-storing renal disease involving both glomerular podocytes and proximal tubular epithelial cells in association with MGRS.

## Case presentation

A 53-year-old Chinese woman was admitted for a 6-month history of foamy urine. Two months before admission, her urinalysis revealed proteinuria 2+ without hematuria. Protein excretion was 2.76 to 3.15 g/24 h. Her serum albumin was 40.1 g/L (normal range: 40–55 g/L), and serum creatinine was 2.20 to 2.50 mg/dl (normal range: 0.50–1.50 mg/dl). Her serum immunoglobulin (Ig) G was 17.2 g/L (normal range: 7.23–16.85 g/L), IgA was 0.59 g/L (normal range: 0.69–3.82 g/L), and IgM was 0.83 g/L (normal range: 0.63–2.77 g/L). Monoclonal IgGκ spike was identified in the serum by immunofixation electrophoresis, and monoclonal IgGκ plus free κ light chain was identified in the urine. Bone marrow aspiration smear revealed 1% plasma cells. CD38, CD138 and CD56 positive cells accounted for 1.13% of bone marrow cells with κ light chain restricted expression as determined by bone marrow flow cytometry. The patient was then referred to our hospital for further evaluation.

She had a 4-year history of hypertension for which she was taking irbesartan. Family history was negative. On admission, the physical examination revealed a blood pressure of 113/65 mmHg, temperature of 36.5 °C, heart rate of 78/min, and respiratory rate of 18/min. No organomegaly was noticed. Other signs were normal.

After admission, urinalysis revealed proteinuria 1.27 g/24 h. The albumin creatinine ratio (ACR) was 751.40 mg/gCr (normal range: < 30 mg/gCr). The urine sediment examination was normal. The urine pH was 5.0, and the specific gravity was 1.007. The urine N-acetyl-β-D-glucosidase (NAG) was 12 U/L (normal range: 0–21 U/L), and α1-microglobulin was 86.1 mg/L (normal range: 0–12 mg/L). Urine glucose was negative. Other laboratory data revealed serum creatinine of 2.34 mg/dl, estimated glomerular filtration rate (eGFR) of 23.00 ml/min/1.73m^2^, serum total protein of 79.5 g/L (normal range: 65–85 g/L), and serum albumin of 42.6 g/L. The sizes of both kidneys were normal. Her serum calcium was 2.39 mmol/L (normal range: 2.11–2.52 mmol/L), phosphate was 1.22 mmol/L (normal range: 0.85–1.51 mmol/L) and the uric acid was 312 μmol/L (normal range: 90–360 μmol/L). Serum liver enzymes were normal. Her white blood cell count was 7.7 × 109 cells/L (normal range: 3.5–9.5 × 109 cells/L), hemoglobin was 148 g/L (normal range: 115–150 g/L) and the platelet count was 205 × 109 cells/L (normal range: 125–300 × 109 cells/L). The prothrombin time was 10.5 s (normal range: 9.0–11.5 s), the activated partial thromboplastin time was 28.9 s (normal range: 26.9–37.6 s) and the plasma fibrinogen level was 3.80 g/L (normal range: 2–4 g/L). She had type 1 cryoglobulinemia with IgGκ. Serum free κ chain was 35.4 mg/L (normal range: 3.30–19.40 mg/L), free λ chain was 16.8 mg/L (normal range: 5.71–26.3 mg/L), and the κ/λ ratio was 2.11 (normal range: 0.26–1.65). Cranial and pelvic bone X-rays did not indicate obvious bone destruction. Echocardiography and abdominal ultrasound were normal. Hepatitis B surface antigen (HBsAg), anti- hepatitis C virus (HCV), anti- human immunodeficiency virus (HIV) and Treponema pallidum antibody (TP-Ab) were all negative. Plasma complement 3 (C3) was 1.240 g/L (normal range: 0.60–1.50 g/L), and complement 4 (C4) was 0.268 g/L (normal range: 0.12–0.36 g/L). Anti-nuclear antibodies, anti-neutrophil cytoplasmic antibodies and anti- phospholipase A2 receptor (PLA2R) antibodies were all negative.

**Fig. 1:**
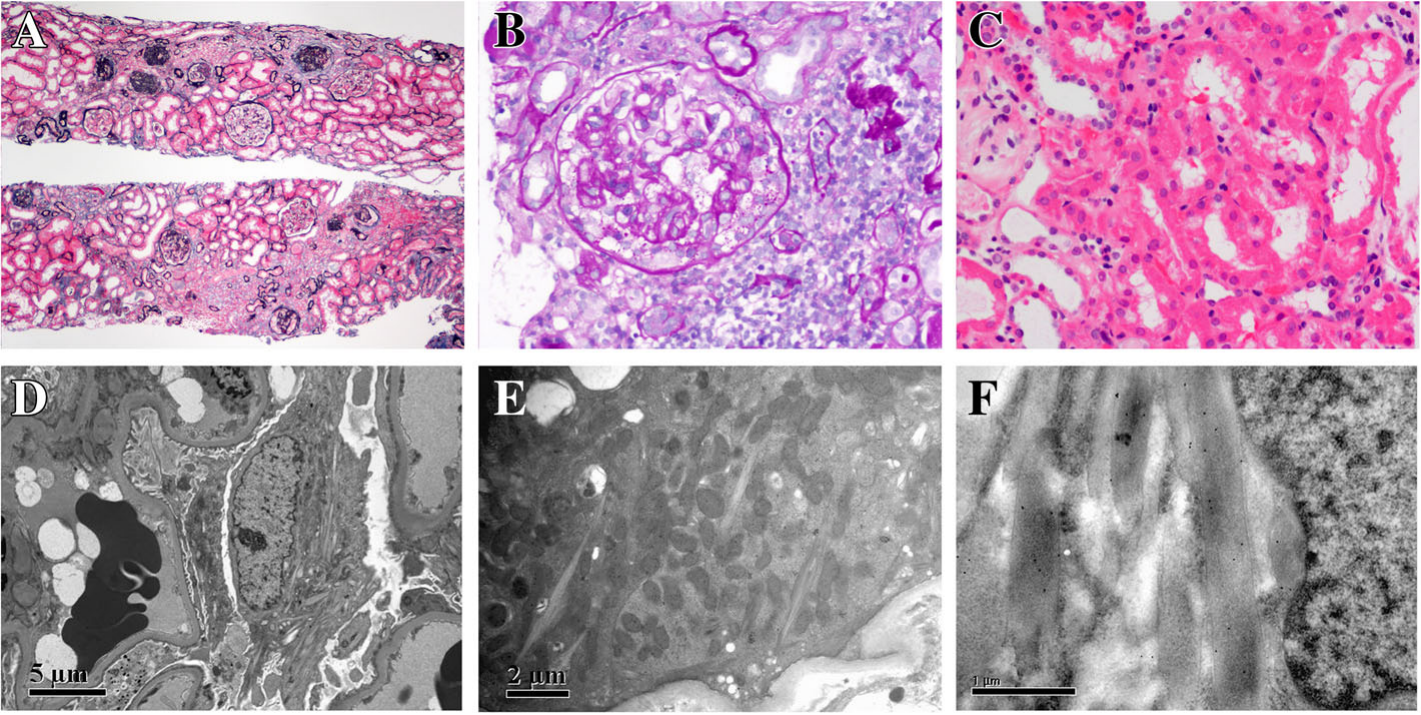
Patient renal biopsy findings. **a** Light microscopy showed some global sclerosis and segmental glomerular sclerosis along with focal tubular atrophy and interstitial inflammatory infiltration. (periodic methenamine silver and Masson trichrome staining, × 40). **b** Glomerular segmental sclerosis with cytoplasmic vacuolization of podocytes. (periodic acid-Schiff staining, × 200). **c** Cytoplasmic vacuolization and eosinophilic granules of proximal tubular epithelial cells. (hematoxylin and eosin, × 200). **d** The rod-like crystals in the cytoplasm of podocytes on EM (× 15,000). **e** Crystals in the proximal tubular epithelial cell on EM (× 12,000). **f** Immuno-electron microscopy (labeled by colloid gold particles with a diameter of 10 nm) indicated κ light chain deposition in the crystals without λ light chain (× 40,000)

MGRS was suspected, but other glomerular diseases accompanied by monoclonal gammopathy of undetermined significance (MGUS) could not be excluded and can only be confirmed by renal biopsy. The patient underwent renal biopsy. Direct immunofluorescence (IF) examination of frozen renal tissue revealed no significant immune deposits and light chains(κ, λ) in the glomeruli, tubules and interstitium. Light microscopic examination showed that 12/29 glomeruli were globally sclerosed and 5/29 glomeruli showed segmental sclerosis with cytoplasmic vacuolization of podocytes (Fig. 1a, b). Other glomeruli were nearly normal. Tubular epithelial cells exhibited focal vacuolization and eosinophilic granules in the cytoplasm and focal loss of brush border with epithelial simplification (Fig. 1c). Tubular atrophy and interstitial fibrosis were minimal. There was mild interstitial infiltration of lymphocytes, monocytes and a few eosinophils. Mild arteriolar sclerosis and intimal fibrosis of the artery were observed. Congo red staining for amyloid was negative. Electron microscopic examination revealed rod- or rhomboid-shaped crystals in the podocytes (Fig. 1d) and proximal tubular epithelial cells (Fig. 1e). The histiocytes did not contain any crystal inclusions. Majority of the podocyte foot processes were effaced. No electron-dense deposits were observed in the glomeruli. Immuno-electron microscopy revealed κ light chain deposition in the crystals without λ light chain (Fig. 1f).

The patient was diagnosed with crystal-storing renal disease involving the podocytes and proximal tubular epithelial cells. She was transferred to the hematological department, and received 4 cycles of CBD (Bortezomib, dexamethasone and cyclophosphamide) protocol chemotherapy. Serum immunofixation electrophoresis still showed IgGκ and urine with IgGκ plus free κ light chain. The patient was considered to be resistant to CBD reatment and switched to Rd. (Lenalidomide and dexamethasone). The patient showed good compliance, and the treatment was well tolerated without clinically significant side effects. The patient was followed up for 12 months until now, and the serum creatinine was approximately 2.26 mg/dl with a proteinuria of 0.3–0.5 g/24 h.

## Discussion and conclusions

Our patient presented with moderate proteinuria and chronic renal failure. Fanconi’s syndrome was insignificant. She had monoclonal IgGκ plus free κ gammopathy that did not meet the criteria of multiple myeloma or lymphoma. Renal biopsy confirmed monoclonal κ-restricted crystal-storing renal disease affecting the podocytes and renal tubular epithelial cells, which confirmed the diagnosis of MGRS.

MGRS-associated renal lesions comprise a wide variety of kidney disorders caused by monoclonal immunoglobulins and their fragments, including light chains or heavy chains. The MIgs can cause renal diseases by direct deposition in the renal tissue in most cases of MGRS or by interfering with complement or coagulation system in rare settings, such as C3 glomerulopathy or atypical hemolytic syndrome secondary to MIgs [1, 16]. Different MGRS manifestations depend on the specific biochemical characteristics of the pathogenic MIg and light/heavy chains involved. Precipitated MIg in the kidney exhibits various forms, including fibrils (amyloidosis, fibrillary glomerulopathy), microtubules (cryoglobulinemic glomerulonephritis, immunotactoid glomerulopathy), and crystals. The crystallization of MIg may occur in the extracellular or intracellular location of renal cells. The former is represented by crystalglobulin-induced nephropathy and cryoglobulinemic glomerulonephritis (GN), which are characterized by intravascular crystals in the thrombi and crystals in the glomerular deposits (mainly subendothelial), respectively. The latter include light chain proximal tubulopathy (tubular cytoplasmic crystals) and crystal-storing histiocytosis (crystals in the cytoplasm of histiocytes or macrophages) [1]. In addition, intracellular crystals have been reported in glomerular endothelial cells, mesangial cells, podocytes, and parietal epithelial cells [3, 4, 14]. However, combined crystalline podocytopathy and tubulopathy have seldom been described.

Our patient had type I cryoglobulinemia, but the renal biopsy exclude cryoglobulinemia-related renal injury. Instead, the renal biopsy revealed crystalline podocytopathy, tubulopathy, and secondary focal segmental glomerulosclerosis (FSGS). Thirteen similar previously reported cases are summarized in Table 1 [3–15]. Most patients (6/13) had a glomerular FSGS pattern or, rarely, collapsing FSGS (especially MM patients treated with pamidronate). The FSGS pattern in these patients is mostly likely secondary FSGS due to crystal deposition-induced podocyte injury, and most of the patients (11/13 patients) had mild to moderate proteinuria, which is similar to our case. However, our patient had a 4-year history of hypertension. The current renal biopsy revealed 12/29 globally sclerosed glomeruli with ischemic change and mild arteriolar and arterial sclerosis, which suggested that hypertension may also have contributed to the renal injury in this patient. In previous reports, ten patients had renal insufficiency and Fanconi syndrome was present in only 2 patients. Majority of the patients (11/13) had myeloma, and all patients including this case were monoclonal IgGκ. Mostly importantly, in crystal-storing renal injury, the IF staining of the light chain on frozen tissue was negative, which may be due to the light chain epitope hiding in the crystal pattern. However, immunostaining for light chains on paraffin tissue after antigenic retrieval and immuno-electron microscopy study can show monoclonal light chain deposition in the crystals, which is very important for the diagnosis.

**Table 1:** Previous reports of crystalline podocytopathy and tubulopathy

Sex/age	Duration of onset to presentation	Clinical renal manifestation	Plasma cell dyscrasia	Glomerular pathology	Crystal distribution	IHC	Treatment	Prognosis
M/29 [12]	12 months	Recurrent proteinuria after two kidney allografts, PCR 6 g/g, SCr 2.3 mg/dl	MGUS→IgG-κ MM	Recurrent FSGS	Podocytes, proximal TEC	IF/IHC:Positive for κ in TEC, λ negative	Bortezomib, lenalidomide, dexamethasone	Lacking
F/66 [11]	During evaluation for back pain	SCr 1.7 mg/dl, Fanconi syndrome, albumin 29 g/L, PCR 3.11μg/mgCr	IgG-κ MM	Non-specific	Podocytes, MC, GEC, TEC, tubular lumen,histiocytes	IF:Positive for κ; negative for λ	Bortezomib, melphalan, prednisolone	Overall improvement in her myeloma related laboratory results
F/52 [10]	Routine health examination	Proteinuria 2.62 g/d, SCr 1.3 mg/dl	IgG-κ MM	FSGS	Podocytes, proximal TEC	IHC: κ positive in TEC, λ negative	Lacking	Lacking
M/45 [9]	Routine annual physical examination	SCr 1.85 mg/dl, proteinuria 7.925 g/d, glycosuria	IgG-κ MM	Collapsing FSGS	Podocytes,MC, TEC	IF/IHC: negative for κ and λ in crystal areas	Therapy, details lacking	2 m later, SCr 1.5 mg/dl, proteinuria 3.627 g/d
M/53 [15]	78 months of MGUS	SCr 1.3 mg/dl, proteinuria 1.18 g/d, albumin 38 g/L	IgG-κ MGUS	Foamy substance in podocytes	Podocytes and TEC	IF: κ TEC positive; λ negative	4 cycles of DF and lenalidomide	SCr returned to 1.0 mg/dl
F/54 [13]	24 months of MM, 19 months of proteinuria	SCr1.0 → 3.9 mg/dl(2 yrs), proteinuria0.3 → 14.4 g/d (2 yrs., pamidronate), albumin 29 g/L	IgG-κ MM	Collapsing FSGS and LCN	Proximal TECs, podocytes,tubular casts	IF: Negative for κ and λ;IHC: Positive for κ, negative for λ	DF, CYC, thalidomide, bortezomib, HCT	SCr 1.8 mg/dl
M/56 [8]	< 1 month	SCr1.2 → 9.2 mg/dl (3 m), proteinuria 5 g/L	IgG-κ MM	NA, ATN	Podocytes, TEC, interstitial macrophages, tubular lumen,BM, urine	IF: Negative for both κ and λ	Vincristine, doxorubicin, DF, HCT	SCr 6.3 mg/dl
F/46 [7]	Unknown	Renal dysfunction	IgG-κ MM	NA	Podocytes, TECs, Interstitial histiocytes	IF: Positive for IgG-κ	Chemotherapy followed by HCT	SCr↓, crystalline- containing podocyte ↓
M/51 [6]	6 months	Bence–Jones proteinuria 1.54 g/L, albumin 41.8 g/L	IgG-κ MM	Nonspecific	Podocytes, GEC, MC, TEC, Interstitial histiocytes,, MCs, hepatocytes and macrophages in liver	NA	Chemotherapy deferred due to lung carcinoma surgery	Died shortly after lung surgery due to multi- organ failure
F/52 [5]	60 months	SCr 1.8 → 2.0 mg/dl (5 yrs), Proteinuria 1.3 → 5 g/d(5 yrs), albumin 34 g/L	IgG-κ MM	3/5 G sclerosed	Podocytes, PEC, TEC, interstitial histiocytes	IF: Negative for κ and λ;IHC: Positive for κ, negative for λ	NA	NA
F/40 [14]	14 months	Proteinuria 14.3 g/d, albumin 30 g/L, SCr 1.8 mg/dl	IgG-κ MGUS	FSGS	Podocytes, PEC, distal TECs, tubular lumina, BM	IHC: Positive for κ, negative for λ	NA	NA
M/75 [4]	60 months of MM	Proteinuria;chronic renal failure	IgG-κ MM	NA	Podocytes, PEC, TEC, interstitial histiocytes, cornea, myeloma cell, choroid plexus	IHC: Positive for κ and γ	NA	NA
M/57 [3]	6 months	SCr 3.2 mg/dl, Proteinuria 2 g/d	IgG-κ MM	FSGS	Podocytes, MC, GEC, PEC, proximal TEC, histiocytes and fibroblasts in the interstitium, synovium and BM	IF: Negative	Cytoxan, carmustine and prednisone, discontinued due to complications	1.5 years later SCr 3.9 mg/dl, died due to cardiac arrest

Abbreviation: *FSGS* focal segmental glomerulosclerosis, *MM* multiple myeloma, *MGUS* monoclonal gammopathy of undetermined significance, *SCr* serum creatinine, *GEC* glomerular endothelial cell, *TEC* tubular epithelial cell, *MC* mesangial cell, *PEC* parietal epithelial cell, *BM* bone marrow, *NA* not available, *HCT* autologous hematopoietic cell transplantation, *ATN* acute tubular necrosis, *DF* dexamethasone, *IHC* immunohistochemistry, *PCR* protein/creatinine ratio, *LCN* light chain cast nephropathy

The exact mechanisms by which monoclonal immunoglobulins form crystals and their different locations in various cells have not been elucidated clearly. Monoclonal immunoglobulins or free light chains are resistant to lysosome enzyme proteolysis due to unique mutations in the variable (*V*) domains of the monoclonal κ light chain that result in substitution of polar residues by hydrophobic residues [17–19]. The undigested light chains formed highly organized crystals within the endolysosomal compartment under certain conditions. There were very rare reports of crystal formation by the λ light chain in the tubular cells and histiocytes [20, 21]. The renal prognosis of crystalline podocytopathy and tubulopathy is variable; most cases progress very slowly, and death is due to extrarenal complications. The treatment of crystal renal disease is debatable. Multiple myeloma patients should be treated with chemotherapy to improve survival, but whether the chemotherapy would prevent renal progression is unclear. However, some previous reports have shown decreased proteinuria and serum creatinine as well as hematological remission after chemotherapy [9, 11, 15], suggesting a benefit of chemotherapy for these patients This case was treated with standard bortezomib followed by lenalidomide-based chemotherapy, and her renal function was stable with a significant decrease in proteinuria after 1 year of follow-up.

This is a rare case of combined crystalline podocytopathy and tubulopathy associated with MGRS. The histological features manifested as FSGS with podocyte crystal formation of κ-light chain restriction as well as tubular injury. The diagnosis was made based on a detailed pathological examination, especially electron microscopy and immuno-electron microscopy. The exact process by which monoclonal immunoglobulins form crystals requires further investigation.

## Abbreviations

ACR: Albumin creatinine ratio
ATN: Acute tubular necrosis
BM: Bone marrow
C3: Complement 3
C4: Complement 4
CBD: Bortezomib, dexamethasone and cyclophosphamide
DF: Dexamethasone
eGFR: Estimated glomerular filtration rate
EM: Electron microscopy
FSGS: Focal segmental glomerulosclerosis
GEC: Glomerular endothelial cell
GN: Glomerulonephritis
HBsAg: Hepatitis B surface antigen
HCT: Autologous hematopoietic cell transplantation
HCV: Hepatitis C virus
IF: Immunofluorescence
IgA: Immunoglobulin A
IgG: Immunoglobulin G
IgM: Immunoglobulin M
IHC: Immunohistochemistry
LCN: Light chain cast nephropathy
MC: Mesangial cell
MGRS: Monoclonal gammopathy of renal significance
MGUS: Monoclonal gammopathy of undetermined significance
MIg: Monoclonal immunoglobulin
MM: Multiple myeloma
NA: Not available
NAG: N-acetyl-β-D-glucosidase
PCR: Protein/creatinine ratio
PEC: Parietal epithelial cell
Rd.: Lenalidomide and dexamethasone
SCr: Serum creatinine
TEC: Tubular epithelial cell
TP-Ab: Treponema pallidum antibody
